# Learning an Intermittent Control Strategy for Postural Balancing Using an EMG-Based Human-Computer Interface

**DOI:** 10.1371/journal.pone.0062956

**Published:** 2013-05-22

**Authors:** Yoshiyuki Asai, Shota Tateyama, Taishin Nomura

**Affiliations:** 1 Okinawa Institute of Science and Technology Graduate University, Okinawa, Japan; 2 Graduate School of Engineering Science, Osaka University, Osaka, Japan; McMaster University, Canada

## Abstract

It has been considered that the brain stabilizes unstable body dynamics by regulating co-activation levels of antagonist muscles. Here we critically reexamined this established theory of impedance control in a postural balancing task using a novel EMG-based human-computer interface, in which subjects were asked to balance a virtual inverted pendulum using visual feedback information on the pendulum's position. The pendulum was actuated by a pair of antagonist joint torques determined in real-time by activations of the corresponding pair of antagonist ankle muscles of subjects standing upright. This motor-task raises a frustrated environment; a large feedback time delay in the sensorimotor loop, as a source of instability, might favor adopting the non-reactive, preprogrammed impedance control, but the ankle muscles are relatively hard to co-activate, which hinders subjects from adopting the impedance control. This study aimed at discovering how experimental subjects resolved this frustrated environment through motor learning. One third of subjects adapted to the balancing task in a way of the impedance-like control. It was remarkable, however, that the majority of subjects did not adopt the impedance control. Instead, they acquired a smart and energetically efficient strategy, in which two muscles were *inactivated* simultaneously at a sequence of optimal timings, leading to intermittent appearance of periods of time during which the pendulum was not actively actuated. Characterizations of muscle *inactivations* and the pendulum¡Çs sway showed that the strategy adopted by those subjects was a type of intermittent control that utilizes a stable manifold of saddle-type unstable upright equilibrium that appeared in the state space of the pendulum when the active actuation was turned off.

## Introduction

Human motor control requires the generation of a sequence of motor commands for muscles that actuate a mechanical body plant with the correct magnitude at the right time to achieve the goal of a motor task. For a novel given goal of a motor task, the brain addresses issues concerning motor planning, state estimation, prediction and motor execution, whereby a task specific control strategy is acquired through motor learning [1], [2], [3]. A simple but critical task is visuomotor tracking, where motor commands are generated based on continuously supplied, usually time-delayed visual feedback information so that the tracking error between a target and an end-effector can be minimized [4]. If a target moves regularly, the brain predicts a subsequent motion, and compensates delay-affected feedback information to better perform the task using an internal inverse and/or forward models [5], [6], [7], [8], [9], [10]. If this is not the case, for example during inherently unstable body dynamics [11], [12] or where there is little knowledge of task-related dynamics [13], control might be compelled to rely on the delayed feedback, leading to an increased risk of delay-induced instability. A typical scenario of such instability during continuous feedback control, such as postural fixation, is a Hopf bifurcation that occurs if a time delay in the feedback is greater than a critical value [14]. Thus, stabilization of unstable body dynamics is another important issue to be addressed by the brain.

In the field of neuroscience, it has been a common view that the brain stabilizes unstable body dynamics using impedance control, which resists destabilizing motion by regulating co-activation levels and thus co-contraction levels of antagonist muscles [15], [16]. Burdet et al [17] and Franklin et al [18] have shown that the central nervous system stabilizes unstable dynamics by learning optimal impedance, in which not all but selected pairs of antagonist muscles associated with the instability are co-activated in a preprogrammed manner. On the one hand, such a feedforward, non-reactive control decreases the risk of delay-induced instability, but on the other hand, a trade-off between stability and high muscle activation arises; increasing impedance enhances the robustness to external perturbations, but co-contraction of muscles increases metabolic cost. For a persistent, basal motor goal such as human upright standing and walking, increase of energy cost could be critical [19]. Internal models representing the relation between motor command and movement might be capable of optimizing such a trade-off not only during voluntary movement, but also during automatic movements such as human standing and walking [20], [21], although there might be substantial differences in neural control mechanisms between discrete and persistent (including rhythmic) movements [22].

Here we critically reexamined the established theory of impedance control as a stabilization strategy in a simple postural balancing task using a novel EMG-based human-computer interface. In this psychophysical experiment, we asked subjects to balance a virtual inverted pendulum with physical parameter values of a typical human body. The pendulum was actuated by a pair of antagonist joint torques that were determined by activations of the corresponding pair of antagonist ankle muscles of subjects standing upright. Positional information of the pendulum was visually and continuously fed back to subjects through a computer screen. The interface developed here provided a motor-task environment similar to stick balancing on a fingertip [23], [24] or on a computer screen [25] as well as balancing a virtual mass in an unstable force-field environment using a manual planar robot [12], [26]. These tasks typically lead to a common motor behavior in which subjects tend to simply keep track of the motion of the inverted pendulum, the stick or the mass. A novel experimental setup used in this study allowed us to address the issue of whether subjects adopt impedance control for stabilizing the pendulum using antagonist muscles.

The motor-task employed in this study raises an interesting frustrated environment at adopting a motor strategy: On the one hand, a large feedback time delay in the sensorimotor loop, as a source of instability, might favor adopting the non-reactive, preprogrammed impedance control, leading to a natural outcome such that subjects adopted impedance control, because it does not require subjects to react to a sequence of falling motions of the pendulum, as traditionally hypothesized in the theory of human postural control [27], [28]. However, on the other hand, the known difficulty of achieving tonic co-activation of antagonist ankle muscles [29], [30], might hinder subjects from adopting the impedance control. Thus, we could also anticipate that an alternative strategy aside from impedance control might emerge under our experimental conditions. This study aimed at discovering how experimental subjects resolved this frustrated motor-task environment through motor learning.

Possible candidates for emerging strategies alternative to the impedance control might be with either time-continuous or discontinuous, intermittent controller. Note that the conventional impedance control is the representative of the continuous control. A controller may or may not be established by acquiring a forward model for state prediction to compensate the destabilizing effect of feedback delay [8], [9], [11], which is often characterized experimentally by a decrease in reaction time through motor learning of anticipation. If subjects adopt a discontinuous, intermittent control strategy in particular, we might observe “on-periods” and “off-periods” of time in activations of the antagonist muscles. In recent years, a variety of (seemingly) different versions of the intermittent motor control have been proposed, mostly during balancing an inverted pendulum. The first version exploits the fact that the state point of the pendulum transiently approaches the upright position along a stable manifold of an unstable saddle-type equilibrium that appears in off-periods during which the active feedback control (i.e. activation of the antagonist muscles) is turned off [31], [32], [33]. This version of intermittent control is effective if a mechanical plant in the absence of active control exhibits saddle-type instability, as during pendulum balancing. It is characterized by the fact that the unstable, but transiently converging saddle-type dynamics during the off-periods are primarily responsible for stabilizing the pendulum, supplemented by the active feedback control during the on-periods. The second version assumes anticipatory ballistic bais control [34], [35], [36], [37], which has been mathematically modeled with a state predictor for compensating a feedback delay [38]. In this case, the anticipatory active feedback control that operates intermittently plays a key role for stabilization, where the intermittency ensures the computational time for the anticipation [39], [40]. The third version considers open-loop periods (off-periods) representing a sensory dead-zone [41], [42], [43], [44], in which a feedback control during the closed-loop periods (on-periods) with appropriate feedback gains is responsible for stabilizing the pendulum. The fourth version is similar to the third one, and has been referred to as the “act-and-wait control,”by which a delay-induced unstable system can be stabilized by an appropriate placement of finite number of poles, if the off-period is larger than the delay time [45], [46], [47]. The fifth version may be termed as the “noise-induced stabilization”with a state-dependent multiplicative motor noise [23], [25], which arises when the delay-affected system is tuned at, or near, the edge of stability and a critical control parameter is stochastically forced back and forth across the boundary. The second to fourth (maybe also the fifth) versions of the intermittent control consider that dynamics during the off-periods do not contribute much to the stability, but to producing intermittent behaviors. Therefore, although there might be connections among those versions of the intermittent control, the first version is a singularity in terms of the stabilization mechanism.

A primary question in this study was whether or not at least some subjects adopted an intermittent control strategy. Moreover, if there were subjects adopted an intermittent control, which version of the intermittent control, among five versions listed above, could better characterize the active control performed by those subjects and the corresponding dynamics of the controlled pendulum?

As we show in this paper, it was remarkable that the majority of subjects controlling the virtual inverted pendulum adopted an intermittent stabilization strategy, and only small population of subjects adopted an impedance-like control. Moreover, characterizations of temporal patterns of muscle *inactivations* and postural sway of the pendulum showed that the intermittent strategy adopted by the subjects was consistent specifically with the one (the first version) that utilizes a stable manifold of saddle-type unstable upright equilibrium in the absence of active feedback control (during the off-periods). Note that multiple strategies could be adopted by different experimental subjects for a single goal of motor task, as pointed by Saha and Morasso [12].

Stochastic fluctuation is unavoidable during postural fixation. Intermittency during arm movement of manual tracking [12], [26], [48], [49] and gaze fixation [50], [51] has been reported. Those fluctuations, including during human standing and stick balancing, often exhibit double power-law behaviors and/or non-Gaussian Lévy-like distribution associated with intermittency [23], [24], [50], [51], [52]. There has been a growing interest in those fluctuations whether they are associated with functional roles [50], [51]. Since a goal of visuomotor tracking is to minimize the tracking error, there might be another trade-off between stability for minimizing the error and flexibility for reacting to motor demands other than the goal of fixation as well as for reducing energetic cost. In this study, we also characterized stochastic sway of the virtual inverted pendulum controlled by subjects in frequency domain, and showed that the postural sway of the pendulum controlled by the intermittent strategy exhibited the power law behavior as reported by Collins and De Luca [52] and as predicted by a mathematical model of the intermittent control [43], [53]. Moreover, we showed that the intermittent control adopted by the majority of subjects was energetically more efficient than the continuous impedance control that was employed by the remaining small population of subjects.

## Methods

### 2.1 Participants

Eleven healthy male subjects with a mean age of 23 years (ranging from 21 and 25 years) participated in the experiment. None of the subjects had any research experiences in human motor control. Experimental procedures of the study were in accordance with the Declaration of Helsinki, and approved by the Ethics Committee on Human Experiments at Graduate School of Engineering Science, Osaka University. All subjects gave written informed consent prior to their inclusion in the study.

### 2.2 Outline of the experiments

**Figure 1:**
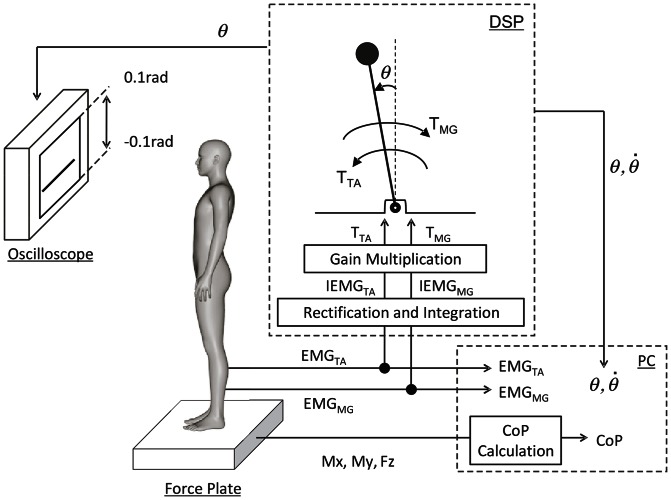
An EMG-based human-computer interface used for virtual balancing. EMGs derived from TA and MG muscles of subjects were fed into a DSP, which were transformed into active torques for balancing a virtual inverted pendulum in the DSP. A vertically moving horizontal bar representing the tilt angle of the pendulum, as a visual feedback information for subjects, was displayed on an oscilloscope screen in front of subjects. EMG signals, force-platform-based CoP motion of subjects and tilt angle of the pendulum were recorded by a PC.

Virtual balancing involved balancing a simulated single inverted pendulum implemented in a digital signal processor (DSP) using an EMG-based human-computer interface (Fig. 1). Subjects were asked to stand upright normally on a force platform barefoot. Motion of the virtual pendulum was displayed in real-time on an oscilloscope screen located in front of subjects as a vertically moving horizontal bar representing the tilt angle of the pendulum. Upward and downward motions of the bar represented forward and backward tilts of the pendulum, respectively. Subjects were instructed to keep the pendulum upright as much as possible under the continuous visual feedback information about the motion of the pendulum. Activation levels of two antagonist ankle muscles of subjects, namely, Tibialis Anterior (TA) and Medial Gastrocnemius (MG) were monitored by electromyography (EMG). The EMG signals were fed into the DSP to obtain integrated electromyograms (iEMGs) of those muscles, which were further transformed into simulated antagonist muscle torques for actuating the pendulum in real-time. Activations of TA and MG muscles generated the simulated muscle torques to pull the pendulum forward and backward, respectively, as in human standing. Subjects modulated their muscle activation levels by altering the tilt angle of their standing body. Subjects were instructed that no joint movements of upper and lower limbs, except the ankle joints, were allowed. Thus, modulation of the body tilt angle was achieved by dorsal and plantar flexions of the ankle joints, inducing small voluntary body sway of subjects in the anterior-posterior direction. It was expected that, when the pendulum tilted forward, subjects also tilted forward to increase the activation level of MG so that the pendulum could be pulled backward, whereas when the pendulum tilted backward, subjects also tilted backward to activate TA muscle so that the pendulum could be pulled forward, implying that subjects might keep track of a sequence of falling motions of the pendulum for balancing the pendulum.

### 2.3 Virtual inverted pendulum

The virtual inverted pendulum was allowed to rotate around a pin joint at the distal end of the pendulum. The joint of the pendulum corresponds to human ankle joints. Dynamics of the pendulum was modeled by the following equation of motion in the sagittal plane.
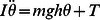
(1)where 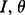, and 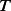 represent the moment of inertia around the joint, the tilt angle from the upright position, and the total torque acting at the joint, respectively. 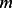, 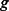, and 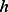 are the mass, the gravitational acceleration, and the length between the joint and the center of mass of the pendulum, respectively. The term 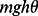 approximates the gravitational toppling torque for small 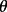. Values of these parameters were similar to those of a typical human body, and fixed as in Table 1.

**Table 1:** Parameter values of the virtual inverted pendulum.

Symbol	Description	Value with Unit
*m*	Mass of the pendulum	60 kg
*g*	Gravitational constant	9.8 m/s^2^
*h*	length between joint and CoM	1.0 m
*I*	Moment of inertia of the pendulum	60 kgm^2^
*K*	Passive elastic coefficient of the joint	0.8 *mgh*Nm/rad
*B*	Passive viscosity coefficient of the joint	15 Nms/rad

The total torque 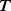 was modeled as 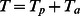, where 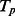 was the passive torque determined by a torsional viscoelastic element at the joint, and 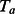 the active torque determined by the muscle activations derived from subjects. The passive torque was implemented as 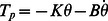, where 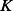 and 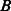 are the passive elastic and viscous coefficients. The active torque was determined by the difference between the antagonist torques as 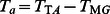, where 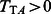 and 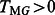 represent, respectively, the forward and the backward bending torques. 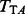 and 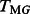 were determined based on the iEMG signals derived from TA and MG muscles of subjects, respectively (see below).

The state space representation of Eq. 1 is as follows.
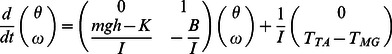
(2)where 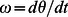. We also use the following abstract formulation of Eq. 2.
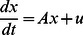
(3)where 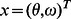, and 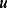 is the active control torque vector. The term 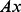 defines the vector field of the non-actively-actuated pendulum with 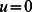.

According to experimental evaluations of the passive ankle visco-elasticity during human quiet standing [54], [55], we set as 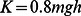 Nm/rad and 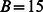 Nms/rad. (The value of 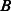 used here might be several times larger than human passive viscosity. However, it was too difficult for most of the subjects to stabilize the pendulum for a smaller value of 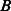.) Thus, 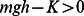, and the upright state 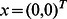 is an unstable equilibrium of saddle type, with stable and unstable manifolds when 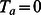 (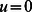) [32]. For the pendulum parameter values used here, the stable and unstable manifolds of 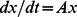 in the 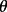-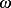 plane are given, respectively, by the straight lines passing through the origin;
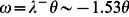
(4)


and
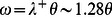
(5)where



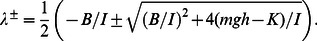
(6)Thus, time constants of the stable and the unstable dynamic modes are 0.65 s and 0.78 s, respectively. Solutions (trajectories) generated by the vector field of 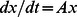 in the 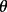-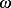 plane exhibit hyperbolic curves that approach the saddle point at 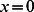 along the stable manifold and fall away from the saddle point along the unstable manifold.

If this mechanical body plant is actively actuated by a continuous delay feedback control that approximates the neural feedback control with a neural transmission delay, a non-negligible risk of the delay-induced instability arises. For example, if we assume a simple proportional and derivative (PD) control with no delay, the unstable saddle point can easily be stabilized asymptotically for a proportional gain greater than 0.2 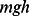 with any positive derivative (velocity) gain. However, if a typical neural transmission delay of 0.2 s is introduced for the PD feedback control, the upright equilibrium can be destabilized by a Hopf bifurcation [14], [56]. Indeed, for small values of derivative gain, the proportional gain of 0.2 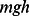 is a critical value, meaning that the proportional gain greater than 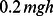 destabilizes the upright equilibrium. See [44] for safety parameter regions of the gains in the delay feedback control, and confirm that the feedback gains (thus the joint impedance) must be carefully tuned for the stabilization, if the continuous feedback control is employed. A reaction time to visually supplied falling motions of the virtual inverted pendulum in this study corresponds to the feedback time delay, and in the later section, we show that it was much larger than 0.2 s, implying that stabilization of the upright equilibrium by the use of continuous feedback control is not easy.

### 2.4 Setup of the EMG-based human-computer interface

EMG signals were monitored from TA and MG muscles of both legs using bipolar surface electrodes separated by 1 cm and a biosignal amplifier (BA1104, TEAC, Inc., Tokyo). Body sway of subjects was recorded as the vertical ground reaction force (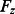), the moment around the mediolateral axis (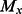) and the moment around the anterior-posterior axis (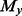) using a force platform (Advanced Mechanical Technology, Inc., USA). 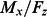 represented motions of Center of Pressure of subjects in the anterior-posterior direction, referred to as CoP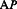. The EMG signals only from the right leg were fed into the DSP (DSK6713IF-A, Hiratsuka Engineering Co. Ltd., Japan) with a sampling frequency of 1 kHz through a 16 bit A/D converter mounted on the DSP, and numerically rectified in the DSP and then processed by the second order Butterworth filter with a cutoff frequency of 12 Hz, yielding the iEMG signals in real-time. The EMG signal from the left leg was not used for the active control of the pendulum, about which subjects were not informed.

In the DSP, dynamics of the virtual inverted pendulum with the active torque 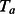 was numerically integrated in real-time using the forward Euler method with a time step of 1 ms. Tilt angle 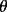 of the pendulum was displayed in a range between −0.1 to 0.1 rad, using the analog oscilloscope placed in front of subjects through a 16 bit D/A converter mounted on the DSP. The iEMG signals, data from the force platform and the tilt angle of the pendulum were recorded by a PC with a sampling frequency of 1 kHz through an 14 bit A/D converter (Keyence, Inc., Japan).

### 2.5 Active torques

Each active torque used for actuating the pendulum was determined as a constant multiple of the iEMG signal. Denoting iEMG of TA and MG muscles as 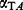 and 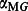, respectively, we determined the active torques as
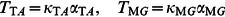
(7)where 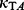 and 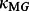 represent the constant gains. These gains were determined individually for each subject prior to the balancing task, because amplitudes of EMG signals for an identical body tilt angle varied across subjects. See Text S1 for details of how we determined the gain constants.

### 2.6 Protocol

The balancing trial started with the initial condition of the pendulum at 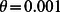 rad and 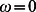 rad/s. When the pendulum's tilt angle departed from a range of 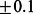 rad, we judged that the pendulum fell down, and counted this as a failure event in the task. If subjects succeeded in keeping the pendulum upright with no failure for 60 s, the trial was considered as a success, or otherwise a failure. After every failure event in a trial, the task was suspended for 3 s, whereby the tilt angle of the pendulum was reset to the initial condition, and then subjects restarted the task until the total performance time span, neglecting the suspended periods, reached 60 s. Initially, all subjects performed twenty trials. If three or more trials were successful, the session was terminated. Otherwise, subjects continued to perform trials until three successful trials were completed, or a maximum of forty total trials was reached.

### 2.7 Data analysis

We examined whether subjects could balance the virtual pendulum by finding appropriate activations of the antagonist ankle muscles through trial and error during the task. In particular, we were interested in examining whether activations of the antagonist muscles were continuous or intermittent. Moreover, we characterized sway motions of the pendulum using the phase space analysis in the 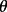-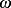 plane and the power spectral analysis.

#### Number of failure events

Number of failure events in every trial was counted for each subject. Since the number of failure events during a successful trial was zero, it was expected that the the number would decrease as subjects adapted to the task.

#### Cross-correlation analysis

We examined the cross-correlation function between tilt angle 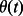 of the pendulum and CoP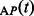 motion of subjects for successful trials. The maximum correlation value and corresponding time-lag were detected, allowing determination of the reaction time for each subject in response to the swaying motion of the pendulum.

#### Activations of the muscles

We examined activation and inactivation patterns of TA and MG muscles. To this end, the active and inactive intervals of each muscle were identified. Active and co-active intervals would be large if the muscles were activated continuously, while they would be small if the muscles were activated intermittently. The second order Butterworth filter with a cutoff frequency of 2 Hz was applied to a given iEMG signal (each of 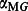 and 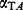) to obtain the smoothed iEMG. If the smoothed iEMG was above a certain threshold curve for a time interval, we defined that interval as an active interval, or otherwise an inactive interval. The threshold curve was defined as follows. We first applied the second order Butterworth filter with a cutoff frequency of 0.02 Hz to the iEMG signal, yielding the slow trend of the iEMG. If the smoothed iEMG was above the slow trend in a time interval, we considered that the muscle activity was high in that interval. In this way, we obtained time intervals in which the muscle activity was high. Then, for each interval with high muscle activity, we further computed the maximum value of the smoothed iEMG. If half of the maximum value was greater than the slow trend, it was defined as the threshold of the interval. Otherwise, the slow trend itself was defined as the threshold of the interval. We determined the threshold curve by performing this procedure for every interval with high muscle activity.

#### Phase plane analysis

Phase plane analysis was performed to examine (1) timings of when inactivations of the active torques occurred and where they were located in the 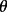-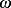 phase plane, and (2) how the pendulum behaved during inactive intervals. Timings and the corresponding locations in the phase plane might characterize a control strategy adopted by subjects. For (1), we specified a sequence of state points in the 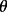-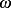 plane when inactivations occurred. For (2), we examined similarity between the motion trajectory of the pendulum that was controlled by subjects and the saddle-type vector field 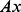 in Eq. 3 in the 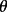-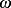 plane. This was because motion of the pendulum should be governed by the saddle-type vector field defined by 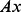 with 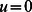 during inactive intervals. Similarity at a time instant 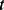 was characterized by direction cosine (DC) between a vector 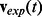 defined as 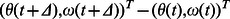 for the pendulum controlled by subjects during success trials and a vector 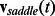 defined as 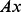 for 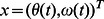 [31]. 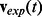 represented a fragment of motion of the pendulum controlled by subjects at time 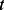, while 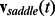 represented a sample of the vector field 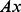 evaluated at the same point where 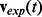 was obtained. DC at time 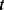 was defined as follows for non-zero 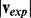 and 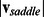.
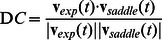
(8)where the operator  represents the inner product. We set 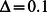 s in this study. Notice that if both TA and MG of subjects were inactivated simultaneously, and hence if there was no active torque actuating the pendulum, the direction cosine would become unity.

#### Power spectral analysis and power-law property

The power spectral density (PSD) of sway motion of the pendulum (tilt angle 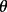) was estimated for successful trials using Fast Fourier Transform (FFT). We characterized double-power-law behavior in the log-log plotted PSD at a low frequency (LF) band of 0.1–.7 Hz and high frequency (HF) band of 0.7–.0 Hz. Slopes of the PSD at LF and HF bands represent the scaling exponents of the power-law behaviors, and they were estimated using linear regressions with the least-square method.

The choice of two frequency bands was based on the observations by Collins and De Luca [52]. They showed that postural sway for healthy adults exhibited power law behaviors by analyzing the mean square displacement of CoP time series data 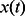 during human quiet standing, defined as 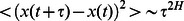, where 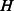 is the scaling exponent (Hurst exponent). More specifically, they showed that CoP sway exhibited three scaling regions with 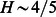, 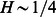 and 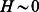, and suggested that those exponents could be interpreted, respectively, as the following behaviors; (1) short-term region (over about 0.7 Hz), corresponding to HF band, where 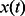 behaved as a positively correlated random walk corresponding to successive fallings due to the gravitational drift, (2) long-term region (about 0.1–.7 Hz), corresponding to LF band, where 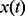 behaved as a negatively correlated random walk corresponding to repulsive movements counteracting the falling movements, and (3) very-long-term region (below about 0.1 Hz) where 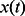 is saturated. Note that the mean square displacement, known also as the two-point correlation function or the stabilogram diffusion plot of the CoP is just an alternative expression of the auto-correlation function of the CoP, which is the inverse Fourier transform of the power spectral density (PSD) of the CoP [57]. Thus, the three scaling regions appear also in the PSD of the CoP as 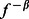 with 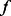 being the frequency, where the two types of scaling exponents are inter-related as 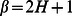 [58]. That is, 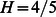 and 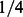 at HF and LF bands corresponds to 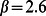 and 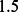, respectively.

#### Energy consumptions

Mechanical energies (powers) consumed by the two antagonist active torques, 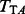 and 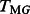 during failed and successful trials were calculated as follows.
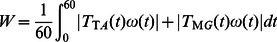
(9)


#### Statistical test

Significance levels of differences were tested using one-way ANOVA in the statistical toolbox of MATLAB (MathWorks, USA). The statistical significance threshold was set basically at 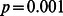 (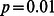 and 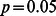 for some cases). Multiple pairwise comparison tests (Tukey's honestly significant difference) were also performed to confirm the results of ANOVA.

## Results

**Figure 2:**
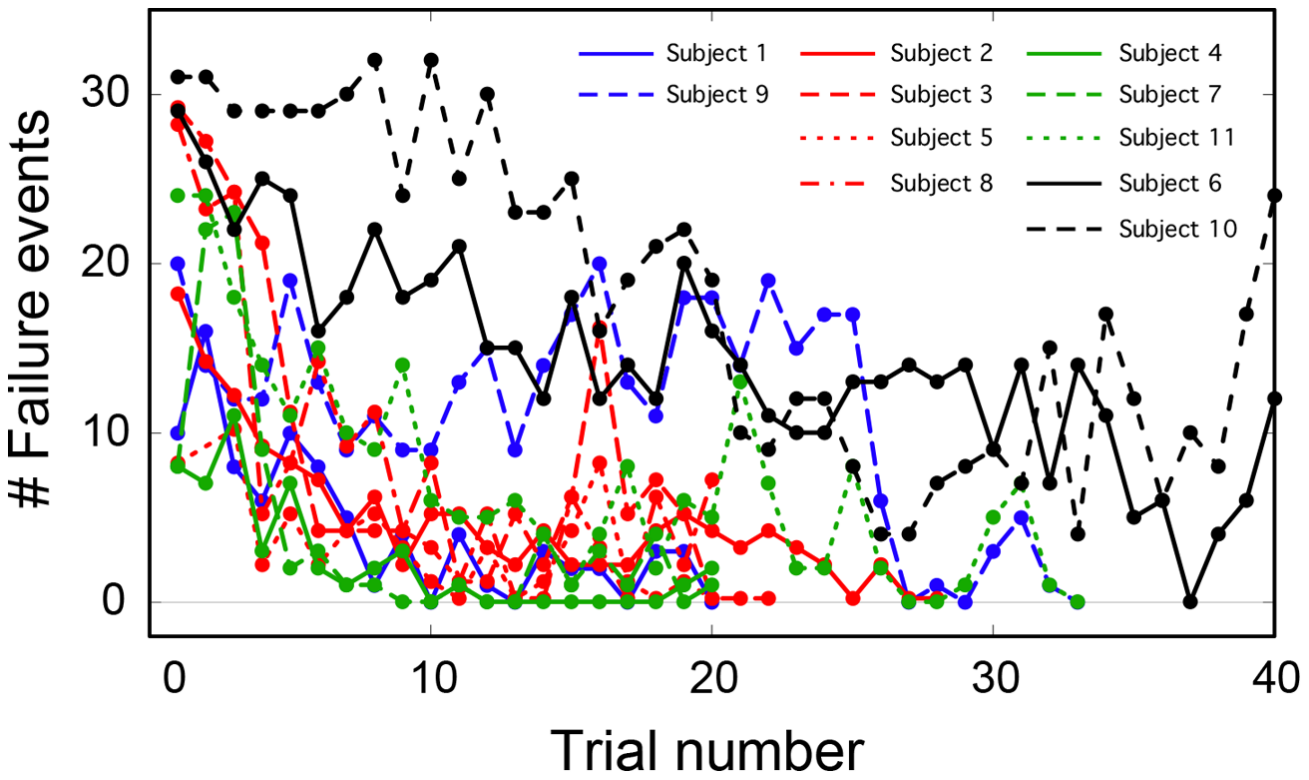
Subject-wise number of failure events within each trial. Each subject was classified into either continuous, one-sided intermittent, or two-sided intermittent type. Blue, red, green curves are for the two-sided intermittent, one-sided intermittent, and the continuous types, respectively. Two subjects could not achieve three success trials within the limited forty trials (black curves).

Nine out of eleven subjects adapted to the task, and succeeded in three or more trials through motor learning. The number of failure events in each trial was roughly a decreasing function of the trial number for each subject (Fig. 2), suggesting that subjects adapted to the task progressively. In most subjects, the number of failure events decreased within the first five to ten trials. Subjects 6 and 10 could not perform three success trials within the limited 40 trials, and they were excluded from further analyses.

Maximum cross-correlations between the tilt angle of the pendulum 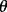 and CoP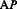 of the subjects were in a range between 0.25 and 0.7 with the average about 0.53, for early (first five; F5) trials and for successful trials. The corresponding time-lags were in a range between −0.24 and −0.45 s with the average about −0.35 s, implying that subjects responded to sway of the pendulum with quite large feedback time delays (reaction times) of about 0.35 s. There were no significant changes in the values of maximum correlation and time-lag between early stage (F5 trials) and late stage (successful trials) of learning. Moreover, there was no clear correlation between the total number of successful trials (i.e., balancing skill) and the maximum cross-correlation, nor between the total number of successful trials and the time-lag, although the time-lag was the smallest in Subject 4 who achieved the highest number of successful trials. These results suggested that subjects did not necessarily acquire a forward model for state prediction (anticipatory reactions) to compensate the delay.

### 3.1 Typical examples and classification of strategies

**Figure 3:**
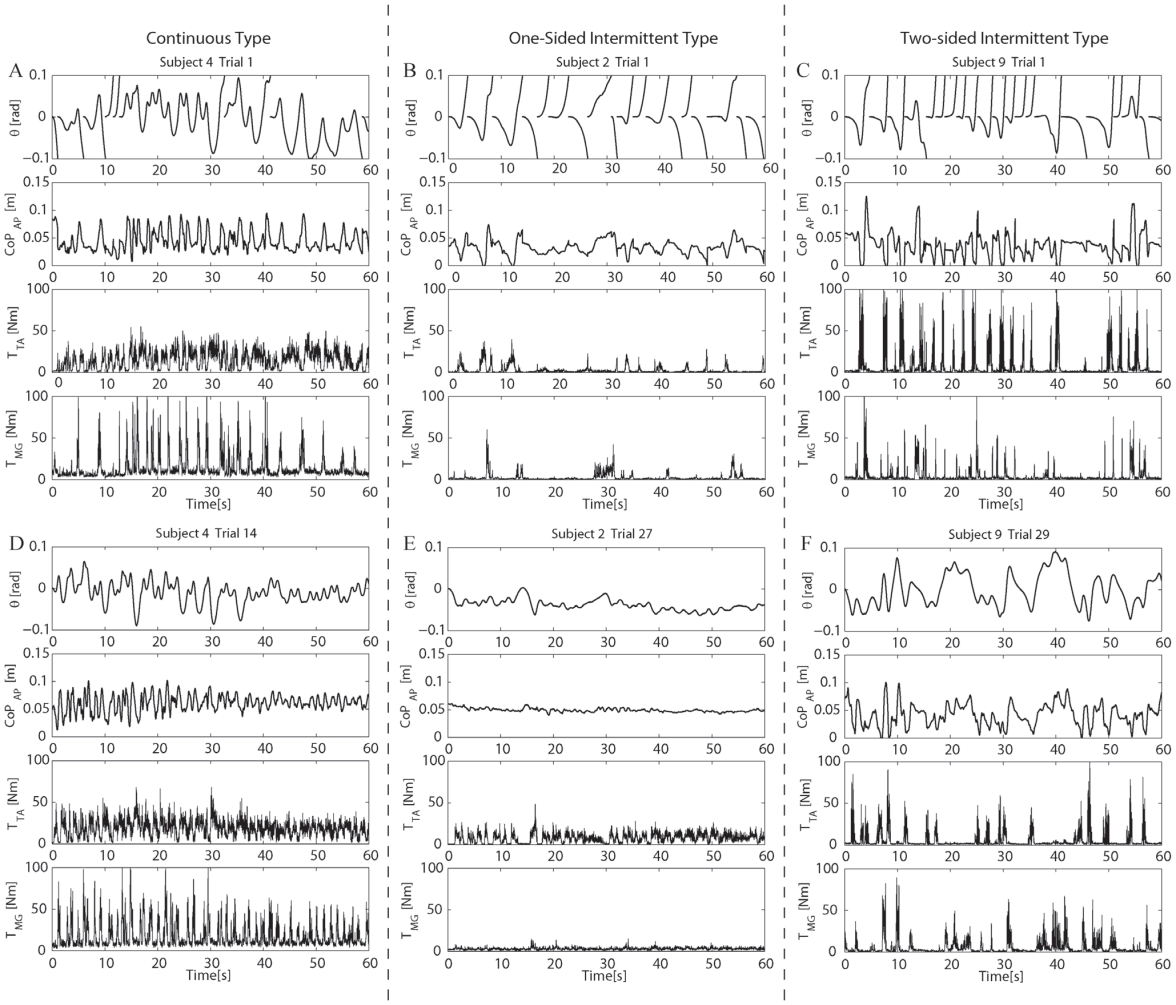
Typical data of failed and successful trials. In each panel, top, second, third and bottom traces show, respectively, tilt angle of the virtual inverted pendulum 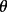, CoP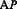 of subjects, active torque 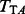 and active torque 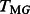. A and D: Failed (#1) and successful (#14) trials in Subject 4 (continuous type). B and E: Failed (#1) and successful (#27) trials in Subject 2 (one-sided intermittent type). C and F: Failed (#1) and successful (#29) trials in Subject 9 (two-sided intermittent type).

Figure 3 shows typical examples of failed and successful trials in three different subjects (Subjects 4, 2 and 9). Indeed, they exhibited representative behaviors for three types of stabilizing strategies (continuous type, one-sided intermittent type, and two-sided intermittent type). Let us introduce those types here for readability of the result section. Through our analysis described in this section, the one-sided and two-sided intermittent types were characterized as the intermittent control, and the continuous type as the continuous impedance-like control. Subject 4 was classified into the continuous type, and Subjects 2 and 9 were classified into the one-sided and two sided intermittent types, respectively.

All of those representative subjects failed to balance the pendulum in the first trial. Subject 4 (continuous type) exhibited only 7 failure events even in the first trial, during which TA was almost continuously activated. Activations of MG were short-lived, but accompanied with a small tonic component (Fig. 3A). Contrastingly, Subjects 2 and 9 (one-sided and two-sided intermittent types) experienced, respectively, 17 and 20 failure events in the first trial (Figs. 3B and C). For each failure event, the pendulum started falling backward (or forward) from the initial condition for a period of time, during which those subjects did not react to the falling motion. After a while, a single delayed impulsive, short-lived for about 1.0 s, TA (or MG) activation from the null activation level was generated in response to the backward (or forward) falling motion until the pendulum reversed the direction and moved across the upright position, where the impulsive activation was terminated. Another delayed impulsive activation of MG (or TA) was then generated to brake the forward (or backward) motion, but it could not prevent the pendulum from falling, leading to a failure event.

Subject 4 (continuous type) succeeded for the first time by the 10-th trial, which was followed by consecutive successful trials. Subjects 2 and 9 (one-sided and two-sided intermittent types), on the other hand, failed repeatedly, but eventually succeeded in three trials (Table 2). For each subject, sway angle 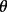 of the virtual inverted pendulum during successful trials was maintained around the upright position (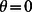). However, they exhibited qualitatively different sway patterns. In Subject 4 (continuous type), sway of the pendulum, and also CoP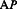 sway of the subject, varied within relatively small ranges (std of 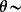
0.025 rad). Moreover, they involved high frequency oscillatory components. Contrastingly, in Subjects 2 and 9 (one-sided and two sided intermittent types), the pendulum as well as the CoP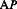 of the subjects exhibited slow sway. Amplitude of the slow sway of 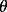 in Subject 2 (one-sided intermittent type) was small (std of 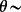
0.015 rad), but that in Subject 9 (two-sided intermittent type) was large (std: 0.036 rad). Sway amplitudes of the pendulum (std of 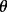) in successful trials were significantly smaller than those in early stage of learning in most of the subjects, including other subjects than those three representative subjects (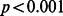 or 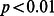), except Subjects 1 and 9 (two-sided intermittent type). Interestingly, average amplitudes for successful trials in Subjects 1 and 9 were larger than those in the remaining subjects.

**Table 2:** Summary of performance of all subjects.

Sub	Total		Succ. trials	Max-corr	Time-lag	Mean std. *θ*
S1 two-int	20	F5 Succ	−10,13,17,20	0.64±0.02 0.66±0.04	−0.22±0.01–0.25±0.01	0.031±0.002 0.025±0.006
S2 one-int	28	F5 Succ	−25,27,28	0.47±0.09 0.57^å^	−0.30±0.52–0.75^å^	0.036±0.001 0.015±0.001†
S3 one-int	22	F5 Succ	−20,21,22	0.41±0.04 0.26±0.12	−0.33±0.03–0.44±0.09	0.036±0.002† 0.014±0.002
S4 cont	20	F5 Succ	−10,12,18	0.61±0.05 0.56±0.11	−0.27±0.01–0.24±0.01	0.041±0.002 0.025±0.003†
S5 one-int	20	F5 Succ	−11,13,18,20	0.21±0.09 0.34±0.01	−0.73±0.12–0.45±0.11	0.027±0.002 0.012±0.001*
S6-	40	F5 Succ	-	-	-	-
S7 cont	20	F5 Succ	−9,10,12,13	0.33±0.08 0.40±0.08	−0.40±0.02–0.43±0.01	0.037±0.002 0.018±0.002†
S8 one-int	20	F5 Succ	−13,14,17	0.52±0.03 0.56±0.06	−0.51±0.06–0.44±0.05	0.039±0.002 0.012±0.001†
S9 two-int	33	F5 Succ	−27,29,33	0.45±0.04 0.69±0.08	−0.28±0.01–0.32±0.01†	0.033±0.001 0.036±0.003
S10-	40	F5 Succ	−37	-	-	-
S11 cont	33	F5 Succ	−27,28,33	0.40±0.03 0.56±0.06	−0.32±0.01–0.26±0.01	0.039±0.002 0.024±0.004*

2nd column: Number of total trials. 3rd column: Successful trial numbers. 4th column: Maximum cross-correlation. 5th column: Time-lag of maximum cross-correlation. 6th column: Mean of standard deviation (std) of 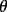. Averages were taken over the first five (F5) trials, and also over successful trials. Significance levels of changes in the indices from F5 to successful trials are indicated by symbols 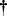 and 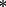 for 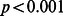 and 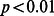, respectively. Max-corr. and Time-lag with 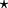 indicate that there was only one trial exhibiting a clear peak in the correlation function. Each subject was classified into either continuous (cont), one-sided intermittent (one-int), or two-sided intermittent (two-int) type.

The active torques (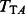 and 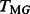) generated by the three representative subjects during successful trials were also different from each other. The active torques of Subject 4 (continuous type) exhibited tonic and high frequency bursts as in the sway waveforms. MG muscle of Subject 2 (one-sided intermittent type) was almost silent, and the activation of TA muscle was more phasic in comparison with Subject 4 (continuous type), and its amplitude was small. Both muscles of Subject 9 (two-sided intermittent type) exhibited sparse and intermittent bursts.

As described here, those preliminary observations across all subjects suggested that there might be three types of intervention strategies; high-frequency, almost continuous interventions as in Subject 4 (continuous type), medium frequency interventions only by one of the two muscles as in Subject 2 (one-sided intermittent type), and low frequency, intermittent interventions as in Subject 9 (two-sided intermittent type).

**Figure 4:**
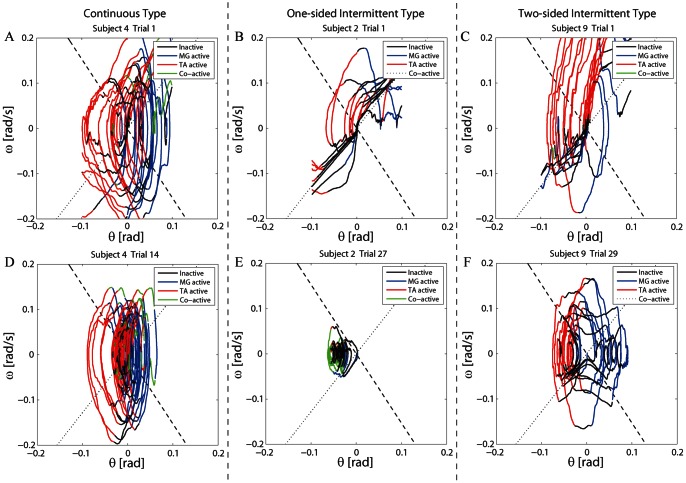
Sway trajectories of the pendulum in the 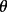-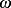 phase plane. Configuration and data used for panels A to F were the same as those in Fig. 3. Each trajectory was segmented into four different groups based on the associated combinations of activations in TA and MG muscles. Back curve segments: Both TA and MG were inactivated (Inactive). Blue segments: TA was inactivated but MG was activated (MG-active). Red segments: TA was activated but MG was inactivated (TA-active). Green segments: Both TA and MG were co-activated (Co-active). Dashed and dotted lines represent the stable and the unstable manifolds of the saddle point defined by Eqs. 4 and 5, respectively, for the virtual pendulum with no active torque.

Sway dynamics of the pendulum were characterized by trajectories in the 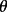-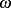 phase plane (Fig. 4). Each trajectory spanning 60 s was segmented and classified into four different groups of segments. Segments of a trajectory in each group were accompanied by a group-specific combination of activation and/or inactivation of TA and MG muscles: Group-1) both TA and MG were inactivated, referred to as the *inactive* segments, Group-2) TA was inactivated but MG was activated, referred to as the *MG-active* segments, Group-3) TA was activated but MG was inactivated, referred to as the *TA-active* segments, and Group-4) both TA and MG were co-activated, referred to as the *co-active* segments.

Trajectories of failed trials in Subject 4 (continuous type) exhibited circular motions around the origin of the plane (Fig. 4A). The associated muscle activation patterns were simple. TA was activated almost continuously when the pendulum was tilted backward to pull the pendulum forward (TA-active segment). When the pendulum was pulled forward across the upright position, TA was inactivated and MG was then activated to pull the pendulum backward (MG-active segment). Transition interval from an inactivation of TA (or MG) to the successive activation of MG (or TA), where both TA and MG were inactivated or co-activated (inactive or co-active segment), was small. Successful trials inherited this feature, but the motions were bounded more tightly around the origin than those in the failed trial (Fig. 4D). Transition intervals were small also in the successful trials, leading to more continuous muscle activations and short time intervals with no muscle activations.

On the other hand, trajectories of Subjects 2 and 9 (one and two-sided intermittent types) in failed trials could be characterized differently by repeated backward and forward slow falling motions along the unstable manifold of the saddle point (Figs. 4B and C). The corresponding segments of trajectories were associated with “slow natural motions”during which those subjects of intermittent types did not activate neither TA nor MG muscle (inactive segments). It was remarkable that this feature of slow natural motions was inherited by their successful trials, and thus the pendulum was not actively actuated for long periods of time (Figs. 4E and F). In successful trials of Subject 2 (one-sided intermittent type), the trajectory circulated almost only in the left-half plane, in which the inactive segments of hyperbolic arcs appeared intermittently (Fig. 4E). Each inactive segment represented a forward motion approaching the origin (the upright position) along the stable manifold and then falling backward along the unstable manifold. In Subject 9 (two-sided intermittent type), the inactive segments formed the upward and downward convex-hyperbolic curves (Fig. 4F) approaching the origin along the stable manifold in one side of the plane, passing across the upright position, and then falling along the unstable manifold in the other side of the plane, suggesting that these natural motions in the inactive segments were governed by the saddle-type vector field of 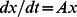 defined in Eq. 3. Moreover, the trajectory circulated within each of left and right sides of the plane, where inactive segments of hyperbolic arcs appeared intermittently as in the case of Subject 2 (one-sided intermittent type). This implied that there were two oscillatory dynamics at left and right sides of the plane, and the state of the pendulum was hopping between them.

Based on the observations described here, we classified nine subjects into either continuous, one-sided intermittent, or two-sided intermittent type. Subjects 1 and 9 were classified into the two-sided intermittent type. Subjects 2, 3, 5 and 8 were classified into the one-sided intermittent type. Subjects 4, 7 and 11 were classified into the continuous type. Those classifications were performed based on several quantitative indices. In this sequel, we describe detailed dynamics during the motor-task with those indices, by which we characterized each type of the control strategy adopted by the subjects.

### 3.2 Activation, inactivation and co-activation of the muscles

**Figure 5:**
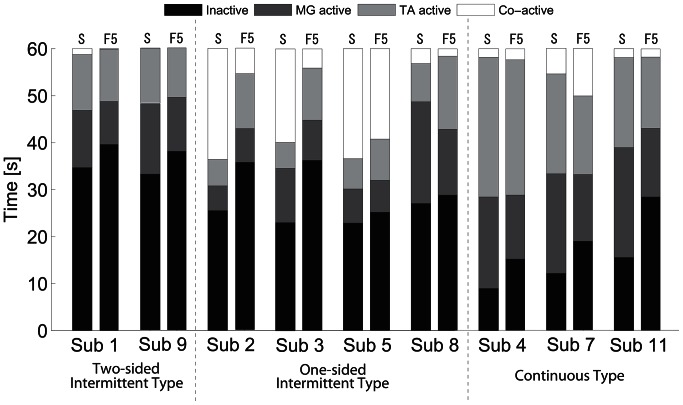
Average durations of inactive, TA-active, MG-active and co-active intervals. See caption of Fig. 4 for definitions of four groups of intervals. Each duration was averaged separately over the first five trials (F5) and the successful trials (S) for each subject. Black bars: Inactive durations. Dark gray bars: MG-active durations. Gray bars: TA-active durations. White bars: Co-active durations.

Time intervals during which TA and/or MG were activated and inactivated were analyzed for all failed and successful trials of nine subjects. For each trial, the whole time span (60 s) was divided into four groups of intervals based on the group-specific combinations of activation and/or inactivation of TA and MG muscles. As in Fig. 4, those four groups were 1) the *inactive* intervals, 2) the *MG-active* intervals, 3) the *TA-active* intervals, and 4) the *co-active* intervals. Average total duration of each interval group was analyzed over the first five (F5) trials and also over all successful trials for each subject to characterize how the subjects adapted to the task (Fig. 5). Note that the sum of average durations of inactive, TA-active, MG-active, and co-active intervals was equal to 60 s.

In all subjects, average inactive durations of the successful trials decreased by a few seconds up to 10 s from those of the F5 trials, and durations with muscle activations increased by the corresponding amounts. However, in Subjects 1 and 9 (two-sided intermittent type), and Subject 5 (one-sided intermittent type), the duration of each group in the successful trials was roughly the same as that in the F5 trials, suggesting that subjects did not necessarily optimize overall frequency of interventions.

#### 3.2.1 Duration of inactive intervals

The inactive intervals during successful trials in the subjects of one-sided and two-sided intermittent types were long. They were the longest for the subjects of two-sided intermittent type (Subjects 1 and 9), occupying more than 50% of the whole time span. The inactive intervals for the subjects of one-sided intermittent type (Subjects 2, 3, 5 and 8) were also long and about 40% of the whole time span.

On the other hand, the inactive intervals during successful trials in the subjects of continuous type were short. In particular, they were only about 10% of the whole time span in Subjects 4 and 7. Indeed, the inactive interval was the shortest in Subject 4 among all subjects.

#### 3.2.2 Activation-balance of two antagonist muscles

TA-active duration was roughly the same as MG-active duration in the subjects of two-sided intermittent type (Subjects 1 and 9) and also in the subjects of continuous type (Subjects 4, 7 and 11), although values of these two durations were different depending on the subject, suggesting that the balancing was achieved by activating the two antagonist muscles equally in the two-sided intermittent and the continuous types. However, two muscles were activated intermittently in the subjects of two-sided intermittent type, whereas they were activated continuously in the continuous type.

In two subjects of one-sided intermittent type (Subjects 2 and 5), the inactive and the co-active intervals dominated the whole time span. However, inspection of the iEMG waveforms for TA and MG revealed that the amplitude of MG activation was quite small, and thus most of the co-active intervals in these cases were in fact largely TA-active intervals. In one subject of one-sided intermittent type (Subject 8), the inactive and MG-active intervals occupied most of the time span. In this way, only one of the antagonist muscles (either TA or MG) was dominantly activated in the subjects of one-sided intermittent type.

### 3.3 Similarity to the saddle-type vector field

**Figure 6:**
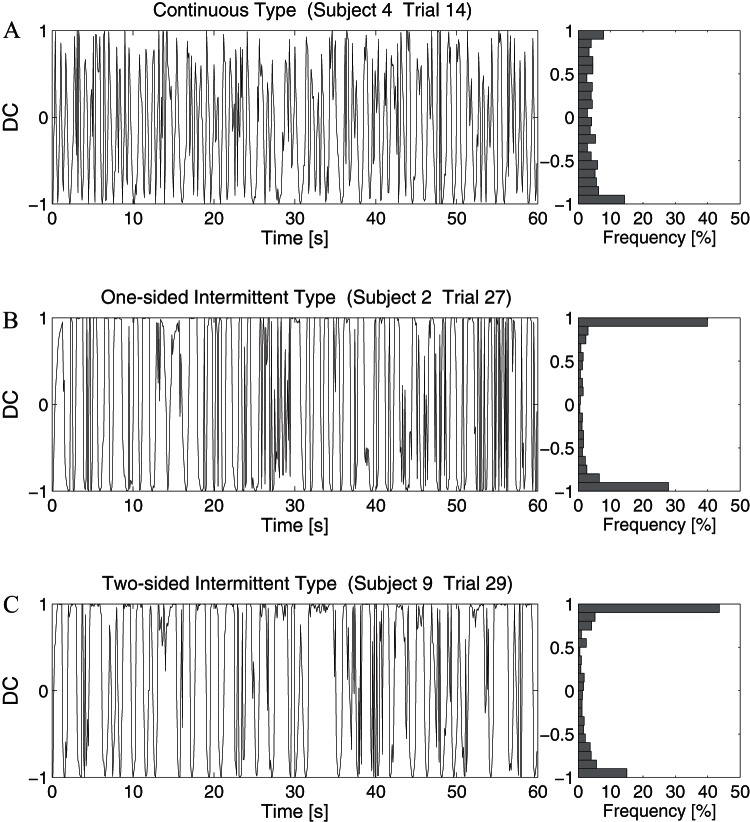
Time-courses and distribution histograms of Direction Cosine (DC) during successful trials. A. Trial 14 of Subject 4 (continuous type). B. Trial 27 of Subject 2 (one-sided intermittent type). C. Trial 29 of Subject 9 (two-sided intermittent type).

Direction cosine (DC) defined by Eq. 8 was calculated to quantify similarity between a phase plane trajectory of the virtual pendulum during each successful trial and the theoretical saddle-type vector field of the pendulum in the absence of active feedback torque. DC-values for failed trials, in particular for F5 trials, were not considered, because DC-values were always close to unity during falling motions along the unstable manifold. If the pendulum moves along the saddle-type vector field in a period of time during a trial, the DC-value in that period of time becomes close to unity. Fig. 6 exemplifies how the DC-value was modulated along time during successful trials in the representative of three types (Subjects 4, 2 and 9).

**Figure 7:**
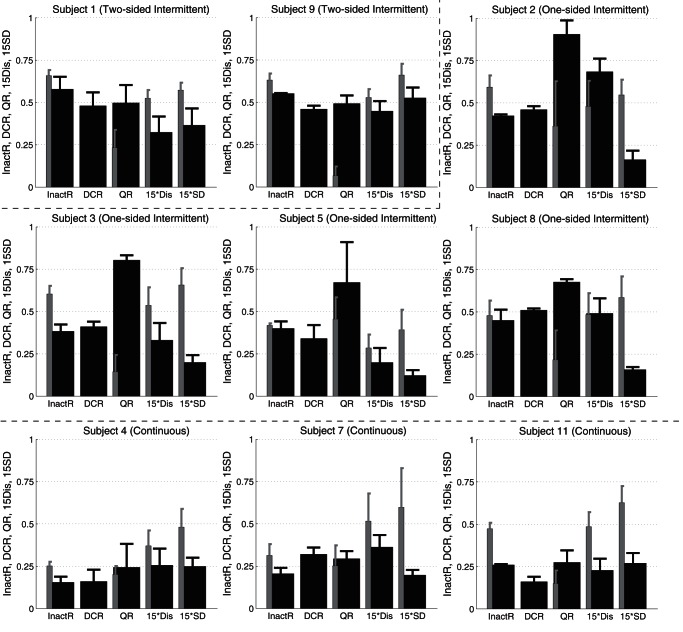
Multiple indices characterizing sway of the virtual inverted pendulum for each subject. In each panel, values of five indices are plotted as thick black bars. From the left-most index, they represent the ratio of inactive interval to the whole trial time span (Inactive-Ratio) denoted as InactR, the ratio of time interval with DC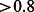 to the whole single trial time span (DC-Ratio) denoted as DCR, the ratio of the number of off-points (an off-point is a state point at a time instant when an inactive interval began) located in specific areas of the 2nd and the 4th quadrants of the phase plane to the total number of off-points (Q-Ratio) denoted as QR, the average of distance between off-points and the 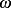-axis in the phase plane denoted as Dis (15 times of Dis-value for scaling: 15Dis), and the average standard deviation of 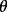-coordinates of off-points denoted as SD (15 times of SD for scaling: 15SD). See text for detailed definitions of indices. Thick black bars are for average values of the indices for successful trials. Thin gray bars are the corresponding values for F5 trials. See text for the meaning of configuration of panels for nine subjects.

We analyzed the total period of time during which the DC-value stayed above 0.8 (i.e., between 0.8 and 1.0) for each of nine subjects. Fig. 7 shows the ratio of the total period of time with DC
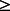
0.8 to the whole time span, referred to as the *DC-Ratio*, averaged over all successful trials for each subject. For example, the average DC-ratios for Subjects 4 of the continuous type, Subject 2 of the one-sided intermittent type and Subject 9 of the two-sided intermittent type in their successful trials were 0.16, 0.46 and 0.46, respectively. Fig. 7 also allows us to associate the DC-Ratios with the durations of inactive intervals. In particular, defining the ratio of the inactive interval duration to the whole trial time span, referred to as *Inactive-Ratio*, we confirmed that the values of DC-Ratio were in agreement with the corresponding values of Inactive-Ratio. That is, the larger the Inactive-Ratio, the larger was the DC-Ratio.

#### 3.3.1 DC-ratio for the continuous type

In the subjects of continuous type, the DC-value showed high frequency oscillations, and was distributed almost uniformly in a range between -1 and 1 (Fig. 6A), indicating that the vector field governing the motion of the pendulum in subject of the continuous type was not saddle-type. This was in agreement with the fact that the subjects of continuous type showed the short inactive durations (Fig. 5). The average DC-Ratio for the subjects of continuous type was 0.22 s. See Table 3.

**Table 3:** Changes in average values of the five indices from F5 trials to successful trials through learning for each subject and for average of each type.

	InactR	DCR	QR	Dis	SD
S1-F5	0.66±0.03		0.23±0.05	0.035±0.002	0.038±0.002
S1-SC	0.58±0.03	0.48±0.01	0.50±0.05*	0.022±0.002*	0.024±0.002*
S9-F5	0.63±0.01		0.07±0.02	0.035±0.002	0.044±0.002
S9-SC	0.55±0.02	0.46±0.03	0.49±0.03†	0.030±0.002	0.035±0.003
two-int-F5	0.65±0.02		0.15±0.03	0.035±0.002	0.041±0.002
two-int-SC	0.57±0.02*[o][c]	0.47±0.03[c]	0.50±0.05†[o][c]	0.025±0.002†	0.029±0.002†[o][c]
S2-F5	0.59±0.03		0.36±0.10	0.032±0.004	0.036±0.002
S2-SC	0.42±0.03†	0.46±0.03	0.90±0.12	0.046±0.005	0.011±0.003†
S3-F5	0.60±0.02		0.14±0.04	0.036±0.003	0.044±0.003
S3-SC	0.38±0.03†	0.41±0.04	0.80±0.05†	0.022±0.004	0.013±0.003†
S5-F5	0.42±0.01		0.46±0.08	0.0190±0.002	0.026±0.003
S5-SC	0.40±0.02	0.34±0.10	0.67±0.09†	0.0132±0.003	0.008±0.003*
S8-F5	0.48±0.04		0.22±0.06	0.0323±0.003	0.039±0.003
S8-SC	0.45±0.05	0.51±0.01	0.68±0.08	0.0327±0.004	0.011±0.004*
one-int-F5	0.52±0.02		0.30±0.04	0.030±0.003	0.036±0.001
one-int-SC	0.41±0.02†[t][c]	0.42±0.03[c]	0.76±0.03†[t][c]	0.027±0.003	0.011±0.002†[t](c)
S4-F5	0.25±0.01		0.20±0.05	0.025±0.003	0.032±0.002
S4-SC	0.15±0.01†	0.16±0.07	0.24±0.04	0.017±0.002	0.017±0.002†
S7-F5	0.31±0.02		0.25±0.04	0.034±0.004	0.040±0.005
S7-SC	0.20±0.02	0.34±0.05	0.29±0.04	0.024±0.004	0.013±0.005*
S11-F5	0.47±0.01		0.15±0.03	0.032±0.002	0.042±0.003
S11-SC	0.26±0.02†	0.16±0.04	0.27±0.04	0.015±0.003†	0.018±0.003*
cont-F5	0.34±0.02		0.20±0.02	0.031±0.002	0.038±0.002
cont-SC	0.19±0.02†[t][o]	0.22±0.02[t][o]	0.26±0.03[t][o]	0.019±0.002†	0.016±0.002†[t](o)

(See text for definition of three types.) Significance levels of changes in the indices from F5 to successful trial were tested for each subject and for each of types. Symbols 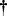 and 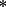 represent that the value of the corresponding index changed significantly with 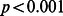 and 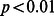, respectively. Parentheses () and brackets [ ] represent the significance levels of difference in the mean values of each index among three types of strategies only for successful trials, respectively with 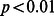 and 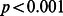. For example, an index-value 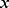 for the one-sided intermittent type (one-int) denoted as “[t](c)”means that the index-value 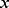 was significantly different from the corresponding index-value for the two-sided intermittent type (two-int) with 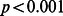, and it was significantly different from the corresponding index-value for the continuous type with 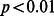.

#### 3.3.2 DC-Ratio for the one and two-sided intermittent types

The DC-value in subjects of one and two-sided intermittent types stayed around unity for much longer periods of time than that in the continuous type, indicating that the pendulum moved along the saddle-type vector field in those long periods of time (Figs. 6B and C). This was also in good agreement with the fact that the inactive intervals in the subjects of one and two-sided intermittent types were long. The corresponding trajectories of the pendulum during inactive intervals could be confirmed by the hyperbolic curved segments shown in Figs. 4E and F. The average DC-Ratios for the subjects of one and two-sided intermittent types were 0.47 and 0.42, respectively, and they were significantly greater than that for the subjects of continuous type (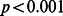). See Table 3 for related statistics.

#### 3.3.3 Inactive-Ratio

In relation to the results shown in Fig. 5, Fig. 7 shows changes in the average durations of inactive intervals (Inactive-Ratio) from F5 trials to the successful trials through learning. In all subjects, the Inactive-Ratio tended to decrease from F5 to the successful trials. The amounts of decrease were significant (either 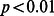 or 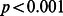) in one third of the subjects, but not in the remaining subjects, confirming that the subjects did not necessarily optimize the intervention frequency (see Table 3 for related statistics).

### 3.4 Inactivation timings of the muscles

**Figure 8:**
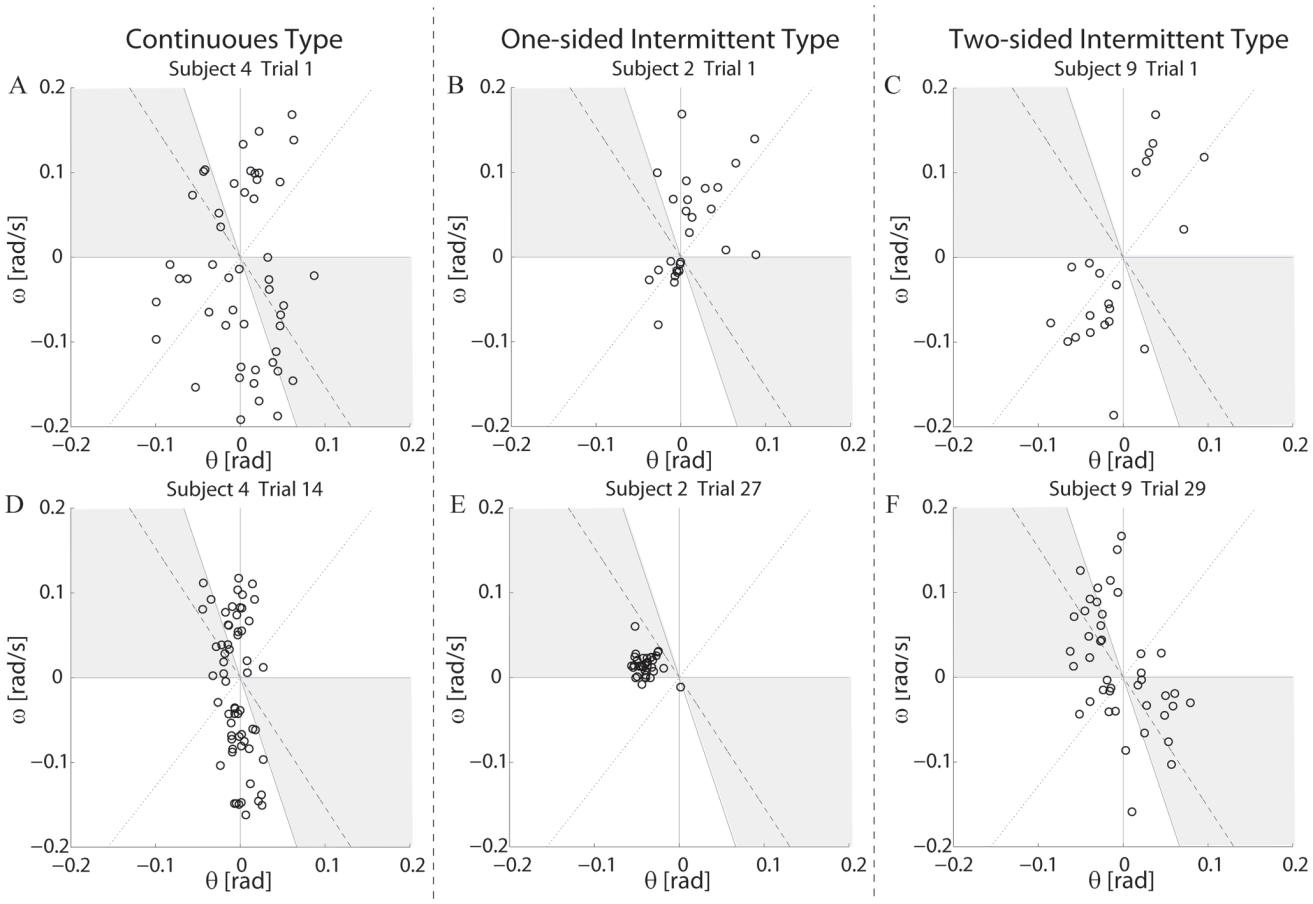
Distributions of off-points in failed and successful trials. An off-point was defined as a state point at a time instant when an inactive interval began. Configuration and data used for panels A to F were the same as those in Figs. 3 and 4. Q-Ratio (Quadrant-Ratio) used to characterize the timing strategy of subjects counted the number of off-points located in the gray triangular areas defined in the second and the fourth quadrants.

In order to characterize the strategies adopted by the subjects, we analyzed changes in the timings when the antagonist muscles were both inactivated. To this end, we specified a location of the pendulum's state point in the 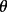-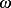 plane, referred to as the *off-point*, at every instant when both TA and MG were inactivated simultaneously. In other words, we specified a sequence of switching-timings from MG-active, TA-active or co-active intervals to inactive intervals. Fig. 8 shows locations of the off-points for failed and successful trials in the representatives of thee types (Subjects 4, 2 and 9). We examined how distributions of the off-points changed through the motor learning, which characterized the state-dependent timing-strategies adopted by the subjects. Since the activation patterns of the subjects of three types were characterized differently even in the first trials, the off-points were also distributed differently in the first trials (Figs. 8A, B and C).

The off-points were distributed widely and almost uniformly in the subjects of continuous type (Fig. 8A), reflecting the fact that they tried to keep the upright position of the pendulum by the high frequency bursts of activations. On the other hand, the off-points were located mainly in the first and third quadrants in the subjects of one and two-sided intermittent types (Figs. 8B and C), reflecting the fact that their delayed response to a falling motion in one direction (forward or backward) was short-lived and terminated when the state point started another falling motion in the other direction along the unstable manifold.

Through learning, the distribution of off-points changed and tended to be close to the 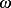-axis in the subjects of continuous type (Fig. 8D). This change was characterized by the distance between each off-point and the 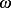-axis. We referred to the distance index as *Dis*. Values of Dis index for the successful trials in the subjects of continuous type were small (Fig. 7 and Table 3), implying that the activated muscle switched immediately before or after the sign of 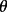 altered. More specifically, the activated muscle switched from MG to TA at an instant when the pendulum moved from forward to backward position, and also switched from TA to MG at an instant when the pendulum moved from backward to forward position, by which the subjects could keep the pendulum's position as close to the upright as possible.

In the subjects of one and two-sided intermittent types, on the other hand, the off-points did not necessarily tend to be close to the 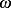-axis. Instead, they moved into the second and/or the fourth quadrants (Figs. 8E and F). Since the off-points in the fourth quadrant were close to the stable manifold of the saddle point of the pendulum in the absence of active control, the state point with the forward-tilted posture and backward velocity moved toward the upright saddle point transiently if no active torques were present. Similarly, the state point in the second quadrant with the backward-tilted posture and forward velocity also moved close to the stable manifold, resulting in the transient approach toward the upright saddle point with no help of muscle activations. These changes in the off-point distribution were characterized by the ratio of the number of off-points located in the triangular areas defined in the second and the fourth quadrants to the total number of off-points. We referred to this ratio as *Q-Ratio* (Quadrant-Ratio). We defined the two triangular areas such that points 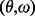 in the areas satisfied the following two inequalities; 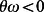 and 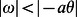, where we set 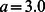. See gray colored triangular areas in Fig. 8. As shown in Fig. 7, values of Q-Ratio for most of the subjects in one and two-sided intermittent types were large. Since these triangular areas did not include the neighborhood of 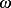-axis, off-points distributed close to the 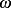-axis, as in the subjects of continuous type, did not contribute much to heightening the Q-Ratio value. Based on this, we used the Q-Ratio value to discriminate the timing-strategy adopted by the subjects of one and two-sided intermittent types from those by the continuous type.

In the successful trials of the subjects of two-sided intermittent type, about half of the off-points located in the triangular areas of the second or the fourth quadrants, and thus the Q-Ratio was about 0.5 (Fig. 7 and Table 3). Since the off-points were distributed relatively widely within the second and the fourth quadrants, the Dis-values of those off-points were also large. Thus, large values of Q-Ratio and Dis indices characterized the timing-strategy of the two-sided intermittent type (Fig. 7 and Table 3).

In the subjects of one-sided intermittent type, the off-points were localized in a small area close to the stable manifold at the second (or the fourth) quadrant. Thus, values of Q-Ratio for the subjects of one-sided intermittent type were quite large (Fig. 7 and Table 3). As confirmed by Fig. 8E, the state point, departed from the off-point, moved forward and approached the upright saddle point along the stable manifold in the second quadrant, and then fell away backward from the saddle point along the unstable manifold in the third quadrant. The activation of TA muscle braked and reversed the falling motion, which brought the state point back to the small area in the second quadrant, generating the slow cyclic trajectory located at the left-half phase plane. In this way, the subjects of one-sided intermittent type exhibited the transient stable motion generated by the saddle-type vector field as in the two-sided intermittent type.

Average Dis-values of the localized off-points for the subjects of one-sided intermittent type were larger than those of the continuous type. However, the difference was not significant, and thus, Dis-value alone could not uniquely characterize dynamics of the pendulum for the one-sided intermittent type, because for some subjects of this type, off-points localized at small areas closer to the 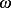-axis as in the continuous type. So, we also quantified a degree of localization of off-points using the standard deviation of 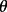-coordinates of the off-points, and referred to this index as *SD*. Because of well-localized off-points in the subjects of one-sided intermittent type, their SD-values were quite small. In this way, we considered that the timing-strategy of the one-sided intermittent type could be characterized by the quite large value of Q-Ratio and the quite small value of SD-value (Fig. 7 and Table 3).

#### 3.4.1 Summary of the timing strategies

Bar plots in Fig. 7 summarize values of the five indices (Inactive-Ratio, DC-Ratio, Q-Ratio, Dis, and SD) that characterized the control strategies of three types. The one and two-sided intermittent types were characterized by the large values of Inactive-Ratio, DC-Ratio and Q-Ratio, in which values of all of these three indices were close to or more than 0.5. On the other hand, the continuous type was characterized by the small values of these three indices that were close to or less than 0.25. The values of SD-index were quite small for the one-sided intermittent type, but relatively large in the two-sided intermittent type, which discriminated between one-sided and two-sided intermittent types. Moreover, the values of Dis-index were relatively large in the two-sided intermittent type. For the subjects in the continuous type, in addition to the small three indices of Inactive-Ratio, DC-Ratio and Q-Ratio, the values of Dis and SD indices were also small.

We confirmed significant differences or similarities in the type-wise average values of those indices, which were consistent with the type-wise characteristics described here. Table 3 summarizes characteristics of all subjects according to the classification of three types.

Fig. 7 and Table 3 describe how the average values of the indices, except DC-Ratio, changed from F5 trials to the successful trials in each subject. There were no clear type-dependent features in the bar plots for F5 trials, except in Inactive-Ratio. As we already observed, the values of Inactive-Ratio for the subjects of one and two-sided intermittent types were large from the early stage (F5 trials) of the session as well as in the successful trials. Although the value of Inactive-Ratio for F5 trials in Subject 11 of the continuous type was also large and the differences in the Inactive-Ratio between F5 and successful trials were significant in some subjects classified in the one and two-sided intermittent types, this feature suggested that the subjects adopted the strategies of one and two-sided intermittent type did not react frequently to falling motions from the initial phase of the motor learning, which was inherited in the late phase of the learning after they acquired the balancing strategies. The values of Q-Ratio were small in almost all subjects for F5 trials, but those values increased remarkably as the subjects of one and two-sided intermittent types learned the balancing task, implying that acquiring this state-dependent optimal timing of muscle inactivation was the key to the motor learning, which enabled those subjects to take advantage of the transient converging dynamics along the stable manifold of the saddle point exhibited by the inverted pendulum with no active control. See Table 3 for statistics.

### 3.5 Power spectral analysis and energy efficiency

**Figure 9:**
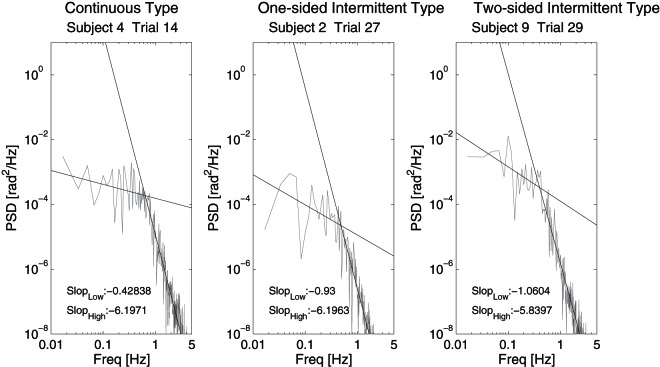
Power spectral density (PSD) functions of sway dynamics (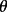) of the pendulum during successful trials. A: Trial 14 of Subject 4 (continuous type). B: Trial 27 of Subject 2 (one-sided intermittent type). C: Trial 29 of Subject 9 (two-sided intermittent type).

Power spectral density (PSD) was estimated for every successful trial. Slopes (scaling factors) at the low and the high frequency bands (LF and HF) of the log-log plotted PSD were calculated to characterize the double power-law behavior in sway of the virtual pendulum. Figs. 9A, B and C show PSDs of the representative subjects of three types.

Table 4 summarizes average slopes at LF and HF bands that were examined separately over the subjects of each type. The average values of slope at LF band for the subjects of two-sided intermittent, one-sided intermittent and continuous types were −0.99, −1.31 and −0.41, respectively. The slopes for two and one-sided intermittent types were significantly steeper than that for the continuous type (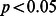). The absolute values of slope at LF band in the intermittent types were close to the corresponding scaling exponent of 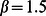 (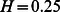) reported by Collins and De Luca [52] in their two-point correlation functions and by Asai et al [32] in their simulation of the intermittent control model.

**Table 4:** Slopes of PSD at LF and HF bands for successful trials and power consumption [W] for F5 and successful trials for each subject and for average of each type.

	LF	HF	Power (F5)	Power (Succ)
S1	−0.96±0.45	−5.84±0.32	1.08±0.12	0.36±0.13*
S9	−1.03±0.16	−6.05±0.33	1.53±0.16	0.61±0.21
two-int	−0.99±0.19{c}	−5.92±0.19	1.30±0.11	0.47±0.13[c]
S2	−1.11±0.70	−6.17±0.10	0.77±0.08	0.27±0.11
S3	−1.23±0.23	−6.51±0.64	2.25±0.29	0.27±0.37*
S5	−1.35±0.27	−5.51±0.32	1.31±0.51	0.23±0.57
S8	−1.54±0.48	−5.94±0.45	2.44±0.74	0.13±0.96
one-int	−1.31±0.14{c}	−5.99±0.14	1.69±0.25	0.22±0.31[c]
S4	−0.20±0.37	−5.80±0.39	2.64±0.28	1.54±0.22
S7	−0.90±0.52	−5.35±0.72	2.85±0.49	0.84±0.55
S11	−0.16±0.99	−5.67±0.57	2.96±0.27	1.16±0.35*
cont	−0.41±0.13{t}{o}	−5.63±0.13	2.81±0.20	1.28±0.20[t][o]

Symbols of 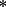 indicate significant difference between F5 and successful trials (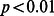). Brackets [ ] and {} represent the significance levels of difference in the mean values of each index among three types of strategies for successful trials, respectively with 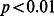 and 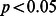. See caption of Table 3.

The average values of slope at HF band in the two-sided intermittent, one-sided intermittent and continuous types were −5.92, −5.99 and −5.63, respectively. There were no significant differences in the slope of the HF band between any two types. The absolute values of slope at HF band in all of three types were about two times larger than the scaling exponent of 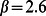 (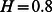) reported by Collins and De Luca [52]. However, the value of about 6.0 was in agreement with the corresponding exponent reported by Asai et al [32] and Nomura et al [53] in their simulation of the intermittent control model.

The steep slopes at LF band for the subjects of one and two-sided intermittent types implied that the long-term-correlated fluctuations were included in the sway motion of the pendulum controlled by the subjects adopted the intermittent control. This was consistent with the fact that sway of 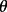 in the subjects of those two types showed slow oscillations, whereas that in the subjects of continuous type did not exhibit slow oscillations, but included high frequency components.

Notice that the average of total power for the continuous type was significantly smaller than that of those for the intermittent types, reflecting the fact that the amplitude of slow oscillation in the continuous type was smaller than those in the intermittent types.

We estimated mechanical energies (powers) consumed by the active torques during F5 and the successful trials (Table 4). For F5 trials, mean powers for the two-sided intermittent, one-sided intermittent and continuous types were 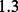 W, 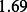 W and 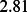 W, respectively, and, for the successful trials, they were 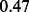 W, 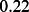 W and 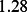 W, respectively. In most subjects, the power was reduced through the learning (but significantly only in three subjects). Moreover, balancing during F5 as well as successful trials for the intermittent types was more energetically efficient than that for the continuous type (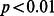). Thus, we concluded that stabilization by the intermittent activations of antagonist muscles for the intermittent types was more energetically efficient than that by the continuous activations for the continuous type.

## Discussion

We examined the theory of impedance control as a strategy for stabilizing unstable dynamics in a simple postural balancing task using a novel EMG-based human-computer interface. We asked subjects to balance a virtual inverted pendulum actuated by a pair of antagonist joint torques that were determined by activations of the corresponding pair of antagonist ankle muscles (TA; Tibialis Anterior and MG; Medial Gastrocnemius) of the subjects standing upright, under the continuous visual feedback information on the pendulum's position.

The motor-task employed in this study raised a frustrated environment. That is, on the one hand, a large feedback time delay in the visuomotor loop is a source of instability, which might favor adopting the non-reactive, preprogrammed impedance control. In our experimental setup, the loop delay was estimated to be about 250 to 450 ms. However, on the other hand, the ankle muscles are relatively hard to co-activate, which might hinder subjects from adopting the impedance control. This study aimed at discovering how experimental subjects resolved this frustrated environment through motor learning. The possible candidates of stabilizing strategies, as an alternative to the impedance control, included 1) to acquire a time-continuous forward model that can predict a current state of the pendulum from a delay-affected feedback information for compensating the delay [8], [9], [11], and 2) a various versions of time-discontinuous intermittent control strategies [32], [36], [42], [46], [23], either with or without a state predictor.

### 4.1 Intermittent use of the stable manifold of saddle-type equilibrium

We showed that the majority of subjects did not adopt the impedance control. Instead, they acquired a discontinuous, intermittent control strategy, in which both of two antagonist muscles were inactivated simultaneously and intermittently, leading to the intermittent appearance of periods of time during which the pendulum was not actively actuated. Let us refer to those periods of time as the “off-periods,”and the remaining periods with muscle activations as the “on-periods.”Based on our analysis of behavioral data, we revealed that a specific type of the intermittent control, among different versions of the intermittent control, was adopted by the majority of subjects through motor learning. In particular, for those subjects, we demonstrated that each of the off-periods began at a specific timing when the state of the pendulum visited a safe neighborhood of the stable manifold of the upright saddle-type equilibrium exhibited by the pendulum in the absence of active control, i.e. during the off-periods. Dynamics of the virtual pendulum described here are consistent with the concept of “gentle taps”for the subjects of the intermittent types, and “brute force”for the subjects of the continuous type, adopted for stabilizing unstable loads [59].

The behavior exhibited by the subjects of (one-sided and two-sided) intermittent types was characterized by the long interval of off-periods occupying nearly half of the task-execution time and the distribution of the state point in the phase plane at every onset of off-period. In this way, these subjects selected not to intervene in the control-free natural motion of the pendulum, during which the state point of the open-loop pendulum close to the stable manifold transiently approached the unstable upright saddle point with no active effort. Of course, after a while, the pendulum started falling away from the saddle point, because the upright saddle point was unstable. Thus, in response to the falling motion, the subjects activated either TA or MG muscle for a short interval of an on-period, by which the velocity 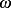 of the pendulum, not the tilt angle 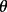, altered the sign, resulted in the state point of the pendulum close to the stable manifold again. Repeating this process could lead to the oscillatory dynamics, typically limit cycles or chaotic oscillations [42], [60], [53]. In this regard, the dynamics observed for the one-sided and two-sided intermittent types were associated with the bistability of the limit cycle dynamics. Dynamics of the one-sided intermittent type correspond to one of the two bistable limit cycle oscillations, located at either left or right half of the phase plane, and those of the two-sided intermittent type to the hopping dynamics between the two limit cycle attractors.

Notice that a role played by the short-lived activations of TA and MG muscles during the on-periods in the subjects of the intermittent types was to alter the sign of the velocity, not the angle. This is consistent with a previous study demonstrating an importance of velocity information for stabilizing upright standing [61]. However, this importance has been interpreted, so far, in terms of continuous-time derivative feedback control, whereas we reconsidered it from a discontinuous-time perspective. As we discuss later in this section, it is worth mentioning that the acceleration information can also be important for the stabilization as demonstrated by Insperger et al [44].

It has been shown that the intermittent control model that utilizes the stable manifold during the off-period can establish robust and flexible stability of the upright posture for single [32] as well as double inverted pendulums [33] actuated at the ankle and/or hip joints, where the intermittent control model alternates between two types of unstable dynamics, depending on the time-delayed physical state of the body pendulum: (1) one mode is characterized by the fact that the active torque is switched off when the pendulum state is located near the stable manifold of the saddle instability; (2) in the other mode, which operates in the remaining time periods, the state is driven by the delayed feedback controller that induces the delay-induced instability. It is remarkable that although both modes are unstable if any of them was adopted permanently, their combination with appropriate switching timings is stable and robust.

In this sequel, we discuss our results in relation to the other possible strategies with time-continuous and discontinuous controllers.

### 4.2 Stabilization by time-continuous controllers

Although the majority of subjects adopted the intermittent control strategy, one third of subjects, classified as the continuous type, achieved balancing by making their muscle activations look as much like co-activated as possible by swaying their body twitchily and continuously. In those subjects, duration of the off-periods was short. This suggested that they adopted a continuous control strategy similar to the impedance control for stabilizing the pendulum. However, since co-contractions of TA and MG muscles were relatively hard to be performed, the control was inevitably to be reactive (responding to the tilt angle 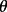 of the pendulum), as opposed to a preprogrammed feedforward control mechanism such as that seen during voluntary arm reaching movements [17], [18]. Indeed, it was more like a bang-bang or a sliding mode control [62], [63], in which two antagonist control commands with opposite actions were switched alternately at the line of 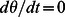 where the sign of the tilt angle was altered.

The outcome of stabilization based on the continuous strategy was surprising, because a continuous and reactive feedback control with a large delay could easily induce instability. However, appropriate tuning of gains for a continuous delay feedback control could avoid instability. See Insperger et al [44] for the stability region in the gain-parameter-space of a continuous delayed proportional and derivative (PD) feedback control of the inverted pendulum. Moreover, they also showed that stabilization by the continuous delayed feedback in the inverted pendulum becomes much easier if a continuous acceleration feedback control is introduced and utilizes a delayed PDA controller. This suggests another possibility that the subjects of our continuous type might utilize the acceleration information for stabilizing the pendulum in the framework of the continuous control.

Yet another candidate for the continuous control strategy is noise-induced stabilization with a state-dependent multiplicative motor noise [23], [25], because variability of motor noise increases with the size of the active motor control signal [64]. Note that, in this case, the controller actuates the pendulum continuously and stochastically, but the resultant dynamics can exhibit intermittency that arises when the delay-affected system is tuned at, or near, the edge of stability and a critical control parameter is stochastically forced back and forth across the boundary [23], in which the term of intermittent is in the sense of intermittency used in statistical physics.

### 4.3 Intermittent anticipatory bias control

Lakie et al [35] studied a balancing of a physical inverted pendulum that was manually controlled by a human hand through a compliant linkage of a spring, where the spring and the motions of the human hand corresponded, respectively, to a compliant tendon and active contractions of muscles. Dynamics of the pendulum in their study might be quite similar to those in the subjects of one-sided intermittent type in our study. Although mechanisms for stabilization in both balancing tasks are likely the same. Lakie et al and related studies [34], [36] suggested that an anticipatory and impulsive control of the bias for the compliant spring was responsible for the stabilization, based on the fact that the reaction times (time-lags in cross-correlations) between motions of the pendulum and the human motion were close to zero in their experiments. In other words, the model of anticipatory bias control might consider a state predictor for compensating the delay-induced instability. It should be mentioned that the intermittent control that utilizes the stable manifold during the off-period determines the active feedback torque based on the delay-affected state of the system, and thus it does not necessarily require a state predictor for robust stabilization. Note, however, that a combination use of the stable manifold during the off-period and an internal model during the on-period is effective as modeled by [31].

Note that changes in the bias (the length of contractile muscles) in the model of bias control might be regarded as a sort of virtual equilibrium-point trajectory [65] that has been considered during human arm reaching movement. However, unlike in the reaching movement, the changes are impulsive in the bias control model during quiet standing. It would be interesting issue to examine whether or not release-timings of the active contractions in the experiment of Lakie et al [35] are located close to the stable manifold during the off-period.

### 4.4 Act-and-wait control: Is intermittency necessary for stabilization?

A main point of discussion here is that stability and intermittent dynamics may or may not be separate issues. In other word, a question is whether or not the intermittency (the off-period) is necessary for stabilization of the inverted pendulum with delay feedback control. As we discussed above, appropriate tuning of the feedback gain parameters in the continuous PD and PDA controllers do not require the off-periods for stabilization [42], [43], [60], [44] even with no help of a state predictor. Of course, the presence of a sensory dead zone in those stable control systems can lead to intermittent dynamics, but it has no effect on stability. In other words, the intermittency may simply be an epiphenomenon or perhaps be beneficial in other ways, e.g. minimize energy requirements as we demonstrated in this paper.

However, there are versions of the intermittent control where the presence of the off-period is critical. The “act-and-wait control” is such an important version of the intermittent control [45], [46], [47]. This control requires a (periodic) sequence of off-periods, and each off-period should be larger than the delay time for stabilizing a delayed feedback control system. This is because, intuitively speaking, destabilizing effect of delay (a memory sequence of states) is forgotten during the off-period, which reduces a problem of stabilization of infinite dimensional delay differential equation into a problem of allocation of finite number of poles. Thus, the intermittent appearance of off-period is critical for the act-and-wait control, as for the intermittent control that utilizes the stable manifold during the off-period.

However, we should note that there is an essential difference between the act-and-wait-control and the intermittent control that utilizes the stable manifold during the off-period. That is, converging dynamics during the on-period of active feedback control is dominantly responsible for the stabilization in the act-and-wait control, whereas transient converging but unstable dynamics during the off-period is important in the intermittent control that utilizes the stable manifold. Stabilization by dynamics during the on-period is also the case for the aforementioned models of intermittent dynamics generated by a sensory dead zone [42], [43]. In this sense, the intermittent control that utilizes the stable manifold is a sort of singularity among various versions of the intermittent control. The present study demonstrated successfully that the stabilization strategy adopted by the majority of subjects was better characterized by and consistent with the specific intermittent control of this sort.

As a computationally positive aspect of the intermittency, Gawthrop et al. [38] have proposed that the intermittency is associated with a computational time necessary for the central nervous system to perform state estimation and prediction. Along this line, Loram et al [39] and van de Kamp et al [40] showed that the reaction time, possibly representing the refractory period in a visuomotor tracking task altered depending on the complexity (the order) of controlled mechanical loads, and claimed that the result could be interpreted by the computational time that induces the behavioral intermittency. However, the results of this study did not necessarily support the existence of delay-compensation that might be achieved by a state estimator and predictor in the central nervous system. Instead, the present study suggests that the subjects in the intermittent types adopted the delay-affected-state-dependent timing strategy that takes advantage of the stable dynamics of the unstable load, which contributes to stabilization of the unstable load.

### 4.5 Energetic aspects

Similar to the theoretical model simulations with the intermittent control [31], [32], the subjects who adopted the intermittent control strategy could achieve flexible upright stabilization, in which no active effort was made to get the state of the pendulum close to the upright state, but the stable dynamics of the unstable saddle point brought the state of the pendulum to the upright state along the stable manifold without any active effort. The role played by the short-lived active torques was just to move the state of the pendulum close to the stable manifold, not to the upright state itself, which could be achieved with less effort and more robustness than forcing the state of the pendulum directly to the upright state. This is why the intermittent strategy was more energetically efficient than the continuous control. Because of the energetic efficiency of the intermittent strategy, the subjects of continuous control type have the potential to change their strategy from the continuous impedance control to the intermittent control, if they continue the trials. Indeed, Franklin et al [13] suggested that, for human arm reaching movement with an unstable force field, feedback gains were up-regulated with increased uncertainty in the knowledge of the dynamics to counteract any errors or disturbances and ensure accurate and skillful movements.

Of course, the total energy requirement is not only the mechanical energy but the amount of electro-chemical energy that must be consumed by the neurons in the central nervous system in order to compute whatever strategy that is being used. Although we showed that the intermittent strategy was more energetically efficient than the continuous impedance-like control from the point of view of mechanical energy, the computational complexity and thus the corresponding energy consumption might be much higher in the intermittent strategy. Thus, the present study should be considered as a basis of further argument that the intermittent-type control is better energetically than the continuous control.

### 4.6 Power-law behavior

Sway of the pendulum controlled by the subjects of intermittent types exhibited the double power-law behavior in the low frequency band (0.1–.7 Hz) that characterizes human postural fluctuation [52], in contrast to the white-noise-like non-scaled flat PSD shape in the subjects of continuous type. The intermittent control model proposed by Bottaro et al [31] and Asai et al [32] can exhibit human-like postural sway with the power-law behavior when it is weakly perturbed by periodic and/or random forcing mimicking the endogenous hemodynamic perturbation [53], [66], suggesting that fluctuation of upright posture is established by the intrinsic oscillatory dynamics of intermittent control, rather than driven by noise.

As discussed by previous studies [32], [43], [53], the double power-law behavior is one of the hallmarks indicating that intermittency is involved in the underlying dynamics. Thus, the power-law behavior observed in the subjects of intermittent types also supported that those subjects adopted the intermittent control strategy.

### 4.7 Remarks and remaining issues

It is important to mention that there are limitations to using a computerized virtual model for postural control. For example, the dynamics for the virtual task are 2-D whereas in the ‘real world’ the dynamics occur in 3-D. Also the computerized virtual model used in this study relies only on visual feedback, whereas the fact that normal subjects can maintain balance with eyes closed, indicating that other forms of sensory information are also important for stabilizing the upright posture.

There are several issues that should be addressed in our future work. As the first issue, one can argue that the variability in outcomes shown in Fig. 2 can be simply due to the fact that the strategies exhibited by the subjects are still in transient learning state, and they are not fixed characteristic of the subjects. In other words, one might ask whether a subject who utilizes the continuous strategy, for example, is always the continuous type strategy user, even when tested the next day or after a few days of practice.

As the second issue, there is not a very good relationship between EMG activity and the force generated by a muscle [67]. Typically, there are electro-mechanical delays between EMG onset and force onset of 60–0 ms, and then between EMG offset and force offset of 120–50 ms. These delays change in complex ways depending on the type of movement that subject is undergoing. Thus, the onset and offset times of EMG in our experimental setup are not necessarily the times when muscle forces were generated.

### 4.8 Conclusion

Most of the previous studies that examined stabilization of unstable dynamics through motor learning assume continuous-time control. Although there might be a considerable difference in the goal of motor task between voluntary arm movements and postural balancing [22], considering a possibility that the central nervous system generates discontinuous motor commands intermittently provides novel opportunities to better understand motor control and dysfunctions. To our best knowledge, the present study first demonstrates a psychophysical experiment for motor learning during persistent movement, which optimizes the timing-strategy by intermittent control.

## Supporting Information

Text S1 **Determining active torques from iEMGs.** Text S1 details how we determined the active torques as constant gain multiples of the iEMG signals from each subject, prior to the balancing task.
(TEX)
