# Correction: Learning an Intermittent Control Strategy for Postural Balancing Using an EMG-Based Human-Computer Interface

**DOI:** 10.1371/annotation/0495873b-830d-498e-8cbd-68af5bb55371

**Published:** 2014-01-14

**Authors:** Yoshiyuki Asai, Shota Tateyama, Taishin Nomura

The file Text S1 was published in an incorrect format. Please view the correct Text S1 file here: 
