# Supplementary material for: Correction: Learning an Intermittent Control Strategy for Postural Balancing Using an EMG-Based Human-Computer Interface
Source: PLoS One. 2014 Jan 14;9(1):10.1371/annotation/0495873b-830d-498e-8cbd-68af5bb55371. doi: 10.1371/annotation/0495873b-830d-498e-8cbd-68af5bb55371 (PMC3891899; doi:10.1371/annotation/0495873b-830d-498e-8cbd-68af5bb55371)
Supplement: Supplementary file 1 [file pone.0495873b-830d-498e-8cbd-68af5bb55371.s001.pdf]

S1. Determining active torques from iEMGs

The active torque T_a actuating the pendulum was modeled as

$$T_a = T_{\mathrm{TA}} - T_{\mathrm{MG}}.$$

Furthermore, we implemented T_{TA} and T_{MG} that were proportional to iEMGs of TA (α_{TA}) and MG (α_{MG}), respectively. Hence,

$$T_a = \kappa_{\mathrm{TA}}\alpha_{\mathrm{TA}} - \kappa_{\mathrm{MG}}\alpha_{\mathrm{MG}} \tag{1}$$

The coefficients κ_{TA} and κ_{MG} were determined individually for each subject prior to the balancing task, where we made use of the fact that α_{TA} and α_{MG} became almost zero, respectively, when subjects modulated the ankle joint angle to tilt their body forward and backward during standing. Let $T_a(\phi, \dot{\phi})$ be the active torque determined by the muscles of subjects and applied to the virtual pendulum when the tilt angle of subjects was ϕ and the corresponding angular velocity was $\dot{\phi}$. Specifically, let ϕ_f and $\dot{\phi}_f$ be the tilt angle and the angular velocity of subjects when subjects tilted forward, by which α_{TA} became (almost) zero and, at that time, $\alpha_{\mathrm{MG}} = \alpha_{\mathrm{MG}}^{\max}$. Similarly, let ϕ_b and $\dot{\phi}_b$ be the tilt angle and the angular velocity of subjects when subjects tilted backward, by which α_{MG} became (almost) zero and, at that time, $\alpha_{\mathrm{TA}} = \alpha_{\mathrm{TA}}^{\max}$. Then, using Eq. 1, we have the following equations for the unknown parameters κ_{TA} and κ_{MG}.

$$\begin{aligned} T_a(\phi_f, \dot{\phi}_f) &= -\kappa_{\mathrm{MG}}\alpha_{\mathrm{MG}}^{\max} \\ T_a(\phi_b, \dot{\phi}_b) &= \kappa_{\mathrm{TA}}\alpha_{\mathrm{TA}}^{\max} \end{aligned}$$

Denoting $T_a(\phi_f, \dot{\phi}_f) = -T_{\mathrm{MG}}^{\max}$ and $T_a(\phi_b, \dot{\phi}_b) = T_{\mathrm{TA}}^{\max}$, we have

$$\kappa_{\mathrm{MG}} = T_{\mathrm{MG}}^{\max}/\alpha_{\mathrm{MG}}^{\max} \tag{2}$$

$$\kappa_{\mathrm{TA}} = T_{\mathrm{TA}}^{\max}/\alpha_{\mathrm{TA}}^{\max} \tag{3}$$

$T_{\mathrm{MG}}^{\max}$ and the corresponding $\alpha_{\mathrm{MG}}^{\max}$, and $T_{\mathrm{TA}}^{\max}$ and the corresponding $\alpha_{\mathrm{TA}}^{\max}$ were determined by a sequence of pre-trial experiments, in which subjects were instructed to perform periodic voluntary body sway with a small amplitude. Subjects were asked to keep upright posture as in the balancing trial, during which $\mathrm{CoP}_{\mathrm{AP}}$ motion of subjects and a sinusoidally moving desired (target) location of $\mathrm{CoP}_{\mathrm{AP}}$, referred to as $\overline{\mathrm{CoP}}_{\mathrm{AP}}$, were displayed as two horizontal bars on the oscilloscope. $\overline{\mathrm{CoP}}_{\mathrm{AP}}$ oscillated sinusoidally around a given baseline position with a frequency of 0.4 Hz and an amplitude of

30 mm. Subjects were intsructed to keep track of $\overline{\mathrm{CoP}}_{\mathrm{AP}}$, i.e., they were asked to perform postural sway so that their own $\mathrm{CoP}_{\mathrm{AP}}$ moved closely with $\overline{\mathrm{CoP}}_{\mathrm{AP}}$ as much as possible.

To determine κ_{MG} using Eq. 2, the baseline position of $\overline{\mathrm{CoP}}_{\mathrm{AP}}$ was varied systematically and optimized so that α_{MG} exhibited periodic bursting synchronously with the sinusoidal sway, by which MG muscle was activated at every anterior peak of $\overline{\mathrm{CoP}}_{\mathrm{AP}}$, and inactivated at every posterior peak of $\overline{\mathrm{CoP}}_{\mathrm{AP}}$. Moreover, α_{TA} was almost zero when $\mathrm{CoP}_{\mathrm{AP}}$ and thus α_{MG} attained their maximum values. After such an optimal tracking sway was established, we detected the i-th anterior peak value of $\mathrm{CoP}^i_{\mathrm{AP}}$ and the corresponding MG activation α^i_{MG} to determine $\alpha^{\max}_{\mathrm{MG}}$, where $i = 1, \cdots, 5$ (i.e., five sinusoidal cycles). In the same way, to determine κ_{TA} using Eq. 3, we detected the i-th posterior peak value of $\mathrm{CoP}^i_{\mathrm{AP}}$ and the corresponding TA activation α^i_{TA} ($i = 1, \cdots, 5$) to determine $\alpha^{\max}_{\mathrm{TA}}$. In this way, we considered average values of α^i_{MG} and α^i_{TA}, respectively, as $\alpha^{\max}_{\mathrm{MG}}$ and $\alpha^{\max}_{\mathrm{TA}}$.

For the i-th anterior peak value of $\mathrm{CoP}^i_{\mathrm{AP}}$ and α^i_{MG}, we obtained the corresponding active torque T^i_{MG} so that we could use Eq. 2. Since we aimed at generating T_{MG} and T_{TA} that could be used to control the virtual inverted pendulum formulated by

$$I\ddot{\theta} = mgh\theta - K\theta - B\dot{\theta} + T_{\mathrm{TA}} - T_{\mathrm{MG}},$$

we associated $T_a(\phi, \dot{\phi})$ with the tilt angle ϕ and the corresponding angular velocity $\dot{\phi}$ of subjects by identifying $(\phi, \dot{\phi})$ of subjects with $(\theta, \dot{\theta})$ of the pendulum. Then, for $\alpha_{\mathrm{TA}} = 0$, thus for $T_{\mathrm{TA}} = 0$, we had

$$T^i_{\mathrm{MG}} = mgh\phi^i_f - K\phi^i_f - B\dot{\phi}^i_f - I\ddot{\phi}^i_f.$$

where ϕ^i_f was the tilt angle of subjects at the i-th anterior peak, which was estimated from the $\mathrm{CoP}^i_{\mathrm{AP}}$ using the relationship between CoP and center of mass of the body pendulum [1]. Similarly, T^i_{TA} at the i-th posterior peak was determined as

$$T^i_{\mathrm{TA}} = -mgh\phi^i_b + K\phi^i_b + B\dot{\phi}^i_b + I\ddot{\phi}^i_b.$$

Finally, based on Eqs. 2 and 3, we estimated κ_{MG} and κ_{TA} as

$$\kappa_{\mathrm{MG}} = \mathrm{mean}\left[T^i_{\mathrm{MG}}/\alpha^i_{\mathrm{MG}}\right] C_{\mathrm{MG}} \quad (4)$$

$$\kappa_{\mathrm{TA}} = \mathrm{mean}\left[T^i_{\mathrm{TA}}/\alpha^i_{\mathrm{TA}}\right] C_{\mathrm{TA}} \quad (5)$$

where C_{MG} and C_{TA} were the subject-wise normalization factors, defined, respectively, as $0.1/\mathrm{mean}\left[\phi^i_f\right]$ and $0.1/\mathrm{mean}\left[\phi^i_b\right]$. These normalization factors were introduced to have a better correspondence between the range of

motion of the pendulum [-0.1,0.1] rad and that of subjects $[\phi_b, \phi_f]$ during tracking sway in the pre-trial experiments. Moreover, maximum amplitudes of T_{TA} and T_{MG} were almost balanced by this normalization.

References

[1] P.G. Morasso, G. Spada, and R. Capra. Computing the com from the cop in postural sway movements. *Human Movement Science*, 18(6):759–767, 1999.
